# NRF2 Activation Inhibits Both TGF-*β*1- and IL-13-Mediated Periostin Expression in Fibroblasts: Benefit of Cinnamaldehyde for Antifibrotic Treatment

**DOI:** 10.1155/2018/2475047

**Published:** 2018-08-07

**Authors:** Yasutaka Mitamura, Mika Murai, Chikage Mitoma, Masutaka Furue

**Affiliations:** Department of Dermatology, Graduate School of Medical Sciences, Kyushu University, Fukuoka, Japan

## Abstract

Systemic fibrosing or sclerotic disorders are life-threatening, but only very limited treatment modalities are available for them. In recent years, periostin (POSTN), a major extracellular matrix component, was established by several studies as a novel key player in the progression of systemic fibrotic disease. In this research, we revealed the involvement of oxidative stress in the expression of POSTN induced by TGF-*β*1 and IL-13 in dermal fibroblasts. We found that the antioxidant cinnamaldehyde activated the NRF2/HMOX1 pathway. Cinnamaldehyde also alleviated TGF-*β*1- and IL-13-mediated production of reactive oxygen species and subsequent POSTN upregulation in dermal fibroblasts. In contrast, NRF2 silencing abolished the cinnamaldehyde-mediated downregulation of POSTN. These results suggest that cinnamaldehyde is a broad inhibitor of POSTN expression covering both TGF-*β*1 and IL-13 signaling. Cinnamaldehyde may thus be beneficial for the treatment of systemic fibrotic diseases.

## 1. Introduction

Periostin (POSTN) is a matricellular protein that is profoundly involved in the development and progression of fibrotic diseases as well as allergy and oncogenesis [1–5]. In *Postn*-deficient mice, bleomycin-induced fibrosis is canceled compared to wild-type control mice [4]. Moreover, the expression of POSTN is upregulated in the lesional skin of scleroderma patients [4, 6, 7]. Serum POSTN levels are also elevated in scleroderma as well as in pulmonary fibrosis [7, 8]. It has also been shown that the downregulation of POSTN attenuates the profibrotic response of hepatic stellate cells [9].

Fibrosis is a characteristic feature in the pathogenesis of a wide spectrum of diseases, including systemic sclerosis, pulmonary fibrotic disorders, renal fibrotic disease, and liver cirrhosis. Although antifibrotic treatments are currently limited [10–18], recent studies have demonstrated that the activation of nuclear factor erythroid-derived 2-like 2 (NRF2) ameliorates profibrotic processes in the lung, kidney, liver, heart, intestine, and skin [19–27]. NRF2 is a master antioxidant transcription factor that upregulates the transcription of various genes encoding antioxidant enzymes such as *HMOX1* and *NQO1* [28, 29]. In parallel with this, the antioxidant phytochemical cinnamaldehyde (CIN) activates NRF2, induces its cytoplasmic-to-nuclear translocation, and upregulates *HMOX1* expression in human keratinocytes and human dental pulp cells [29, 30]. In addition, the CIN-containing traditional herbal *Kampo* medicine *Keishi-bukuryo-gan* inhibits collagen production in cultured fibroblasts obtained from scleroderma patients [31]. Moreover, this kind of *Kampo* medicines is effective at reducing total skin thickness score in some scleroderma patients [32]. However, to the best of our knowledge, no reports have addressed whether NRF2 affects POSTN expression.

The expression of POSTN is upregulated by two distinct cytokine-mediated pathways, namely, transforming growth factor-*β*1 (TGF-*β*1) and interleukin-13 (IL-13)/IL-4 [9, 33, 34]. In this study, we demonstrated for the first time that CIN-mediated NRF2 activation inhibited the POSTN expression induced by TGF-*β*1 as well as by IL-13 signaling. The dual inhibitory actions of CIN-NRF2 activation suggest that CIN-containing medicaments could have therapeutic benefit for treating fibrotic disorders.

## 2. Materials and Methods

### 2.1. Reagents and Antibodies

Cinnamaldehyde was obtained from Sigma-Aldrich (St. Louis, MO, USA) and dissolved in dimethyl sulfoxide (DMSO; Sigma-Aldrich) or in 100% ethanol (Wako, Osaka, Japan) at a concentration of 5 mM. Anti-NRF2 rabbit polyclonal antibody (H-300) was obtained from Santa Cruz Biotechnology (Dallas, TX, USA), and horseradish peroxidase- (HRP-) linked anti-rabbit IgG antibody (#7074) was purchased from Cell Signaling Technology (Danvers, MA, USA). For Western blotting analysis, an anti-NRF2 rabbit polyclonal antibody (PA5-27882; Thermo Fisher Scientific), anti-HMOX1 rabbit polyclonal antibody (ab137749; Abcam), anti-NQO1 mouse monoclonal antibody (ab28947; Abcam), and anti-GAPDH (14c10) rabbit monoclonal antibody (#2118; Cell Signaling Technology) were used.

### 2.2. Cell Culture

Normal human dermal fibroblasts (NHDFs) were purchased from Lonza (Basel, Switzerland) and were maintained in accordance with the vendor's recommendations. They were pretreated with the indicated concentration of CIN for 30 min and then treated with 5 ng/mL TGF-*β*1 or IL-13 (PeproTech, Rocky Hill, NJ, USA) for 48 h. For some experiments, NHDFs were treated with CIN (25 *μ*M) for 6 h. Then, RNA extracts were subjected to quantitative reverse transcription PCR (qRT-PCR). For knockdown experiments, NHDFs were cultured with small interfering RNA (siRNA) of NRF2 for 24 h, followed by stimulation with 5 ng/mL TGF-*β*1 or IL-13 for 48 h. Then, RNA extracts were subjected to qRT-PCR. For a reconstitution experiment, NHDFs were cultured with siRNA of NRF2 for 24 h, followed by pretreatment with 25 *μ*M CIN for 30 min and then stimulation with 5 ng/mL TGF-*β*1 or 50 ng/mL IL-13 for 48 h. RNA extracts and the supernatants were subjected to qRT-PCR or ELISA, respectively.

### 2.3. Immunofluorescence

NHDFs were placed on an eight-well *μ*-slide (Ibidi, Munich, Germany) and at subconfluence were treated with 25 *μ*M CIN for 3 h. Then, the cells were washed with PBS, fixed with acetone for 10 min, and blocked with 5% (*w*/*v*) bovine serum albumin in PBS for 30 min. Samples were incubated with a primary rabbit anti-NRF2 antibody or control IgG (Abcam, Cambridge, UK). Specific binding was detected using an HRP-conjugated goat anti-rabbit antibody, followed by tyramide labeling with green-fluorescent Alexa Fluor 488 (Molecular Probes, Eugene, OR, USA), in accordance with the manufacturer's protocol. Samples were covered with UltraCruz™ mounting medium containing 4′,6-diamidino-2-phenylindole (DAPI) (Santa Cruz Biotechnology) to preserve fluorescence. The fluorescence images were acquired using an EVOS FL cell imaging system (Life Technologies, Carlsbad, CA, USA).

### 2.4. Detection of Reactive Oxygen Species (ROS) Production

NHDFs were cultured in a 48-well plate for 24 h. Then, the cells were washed with PBS and stimulated with 20 ng/mL TGF-*β*1 or 50 ng/mL IL-13 (PeproTech) for 20 min. Detection of ROS production was performed under a microscope using an Image-IT™ LIVE Green Reactive Oxygen Species Detection Kit (Thermo Fisher Scientific, Rockford, IL, USA). Briefly, the oxidation-sensitive dye 5-(and-6)-carboxy-20,70-dichlorodihydrofluorescein diacetate (carboxy-H_2_DCFDA) (Molecular Probes) was used to quantify ROS levels in live cells. Cells were incubated with Dulbecco's phosphate-buffered saline (DPBS; Thermo Fisher Scientific) with calcium chloride and magnesium chloride containing carboxy-H_2_DCFDA (25 mM) for 25 min at 37°C. Then, the medium was changed to DPBS containing Hoechst 33342 (Thermo Fisher Scientific) as a blue-fluorescent, cell-permeant nucleic acid stain. The fluorescence images were acquired using an EVOS FL cell imaging system (Life Technologies).

### 2.5. Knockdown of mRNA by siRNA

siRNA oligonucleotides were purchased from Dharmacon/GE Healthcare (Lafayette, CO, USA). Cells were transfected with 5 nM ON-TARGET plus siRNA for NRF2 or control for 24 h in the presence of RNAiMAX reagent (Thermo Fisher Scientific), in accordance with the manufacturer's instructions. Silencing of target genes was confirmed by qRT-PCR.

### 2.6. Quantitative Real-Time Polymerase Chain Reaction (qRT-PCR)

Total RNA was isolated using RNAiso PLUS (Takara Bio, Otsu, Japan) and reverse-transcribed with the RNA-direct™ Realtime PCR Master Mix (Toyobo, Osaka, Japan). The PCR reactions were performed on a StepOnePlus Real-Time PCR System (Life Technologies) using the THUNDERBIRD SYBR qPCR Mix (Toyobo). Threshold cycles of primer probes were normalized to a housekeeping gene (glyceraldehyde-3-phosphate dehydrogenase (GAPDH)), and relative values were calculated. Primers for qRT-PCR are described in Table 1.

### 2.7. Enzyme-Linked Immunosorbent Assay (ELISA) for Periostin Production

ELISA for POSTN was performed using two different anti-POSTN Abs, SS18A (the capture antibody) and SS17B (the detection antibody) (Shino-Test, Tokyo, Japan), as previously described [35].

### 2.8. Western Blotting Analysis

Western blotting was performed as previously described [36]. The antibodies (Abs) used in this study were against NRF2 (1 : 1000 dilution), HMOX1 (1 : 1000 dilution), NQO1 (1 : 1000 dilution), and GAPDH (1 : 1000 dilution). The HRP-conjugated anti-mouse or anti-rabbit IgG antibody (Cell Signaling Technology) was used as a secondary antibody.

### 2.9. Statistical Analysis

Data are presented as mean ± SD. Statistical analyses were performed using Prism 5.0 software (GraphPad Software, La Jolla, CA, USA). The significance of differences was assessed using an unpaired one-tailed or two-tailed Student's *t*-test. Values of *P* < 0.05 were considered statistically significant.

## 3. Results

### 3.1. CIN Activates NRF2 Signaling and Induces HMOX1 Production in Dermal Fibroblasts

**Figure 1:**
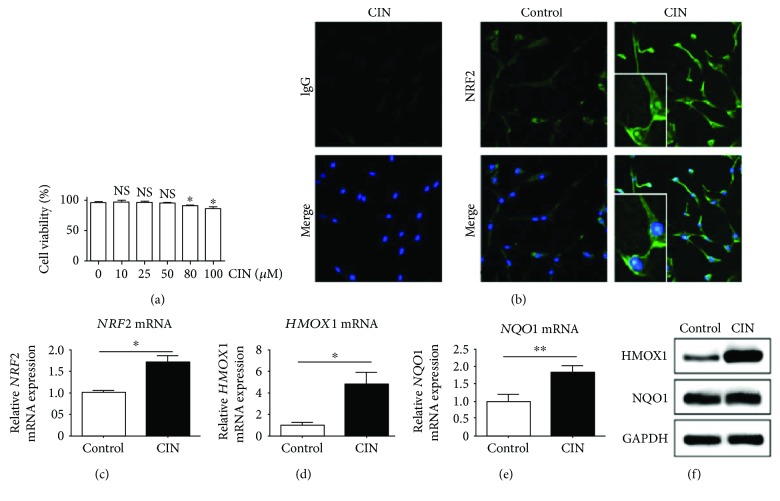
Activation of the NRF2/HMOX1 and NQO-1 pathway by cinnamaldehyde. (a) Effects of cinnamaldehyde (CIN) on the viability of NHDFs. (b) NHDF cells were treated with DMSO (control) or CIN (25 *μ*M) for 3 h and fixed. Cells were then stained with the anti-NRF2 antibody or control IgG (green) and DAPI (blue) and visualized by fluorescence microscopy. (c–e) NHDF cells were treated with DMSO (control) or CIN (25 *μ*M) for 6 h, after which total RNA was extracted. (c) *NRF2* mRNA expression, (d) *HMOX1* mRNA expression, and (e) *NQO1* mRNA expression are depicted. (f) NHDF cells were treated with 100% ethanol (control) or CIN (25 *μ*M) for 24 h, after which whole cell lysates were extracted. The expression of HMOX1, NQO1, and GAPDH was depicted. The values were adjusted by *GAPDH* expression. The same experiments were performed three times. ^∗^*P* < 0.05, ^∗∗^*P* < 0.01. NS: not significant.

CIN induces the cytoplasmic-to-nuclear translocation of NRF2 in human keratinocytes and acts against oxidative stress [29]. Here, we first examined the effects of CIN on the viability of cultured fibroblasts. CIN at concentrations up to 50 *μ*M did not affect the viability of fibroblasts, as determined by the trypan blue dye exclusion test (Figure 1(a)). As previously demonstrated in keratinocytes [29], CIN also induced the cytoplasmic-to-nuclear translocation of NRF2 in dermal fibroblasts (Figure 1(b)). In addition, CIN induced upregulation of the mRNA expression of NRF2 (Figure 1(c)). In accordance with NRF2 activation, CIN upregulated *HMOX1* and *NQO1* expression at the mRNA level, but only HMOX1 expression at the protein level (Figures 1(d)–1(f)). These results demonstrate that CIN activates the primarily NRF2/HMOX1 antioxidant pathway in fibroblasts.

### 3.2. CIN Inhibits POSTN Expression Induced by TGF-*β*1 and IL-13 in Dermal Fibroblasts

**Figure 2:**
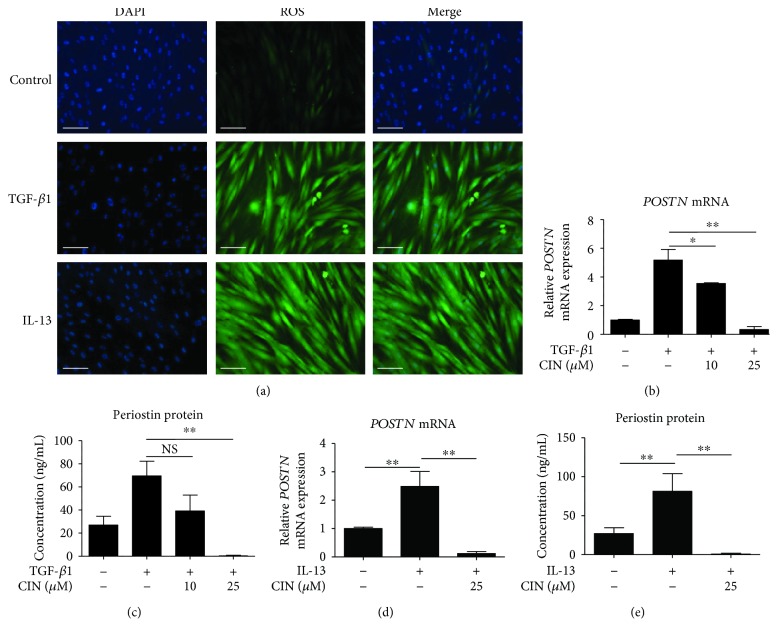
Effects of cinnamaldehyde on TGF-*β*1- and IL-13-induced expression of periostin mRNA and protein in dermal fibroblasts. (a) NHDFs were stimulated with 20 ng/mL TGF-*β*1 or 50 ng/mL IL-13 or unstimulated (control) for 20 min. Cells were incubated with carboxy-H_2_DCFDA for 25 min, after which ROS production was visualized by fluorescence microscopy. Scale bars, 200 *μ*m. (b, c) Effects of cinnamaldehyde (CIN) on TGF-*β*1-induced expression levels of periostin (POSTN) mRNA and protein. (d, e) Effects of CIN on IL-13-induced expression of *POSTN* mRNA and protein. NHDF cells were precultured with the indicated concentration of CIN for 30 min and then treated with 5 ng/mL TGF-*β*1 or IL-13 or left untreated for 48 h. Expression levels of *POSTN* mRNA and protein in the cell supernatants are depicted. The values were adjusted by *GAPDH* expression. The same experiments were performed three times. ^∗^*P* < 0.05, ^∗∗^*P* < 0.01. NS: not significant.

In previous studies, it was demonstrated that oxidative stress induces the expression of POSTN in myocardial fibroblasts and human umbilical vein endothelial cells [37, 38]. Therefore, to further evaluate the antioxidant activities of CIN, we investigated whether CIN is associated with the induction of POSTN expression in dermal fibroblasts. First, we investigated whether TGF-*β*1 and IL-13 induce the generation of intracellular ROS. Compared with unstimulated cells, both TGF-*β*1 and IL-13 clearly induced intracellular ROS formation in NHDF cells (Figure 2(a)). As shown in Figures 2(b)–2(e), both TGF-*β*1 and IL-13 upregulated the gene and protein expression of POSTN in dermal fibroblasts. At a noncytotoxic concentration, CIN inhibited the TGF-*β*1-induced upregulation of the gene (Figure 2(b)) and protein (Figure 2(c)) expression of POSTN. It also inhibited the IL-13-induced upregulation of POSTN expression at the mRNA and protein levels (Figures 2(d) and 2(e)).

### 3.3. The Involvement of NRF2 in the Regulation of the mRNA Expression of Periostin Induced by TGF-*β*1 and IL-13

**Figure 3:**
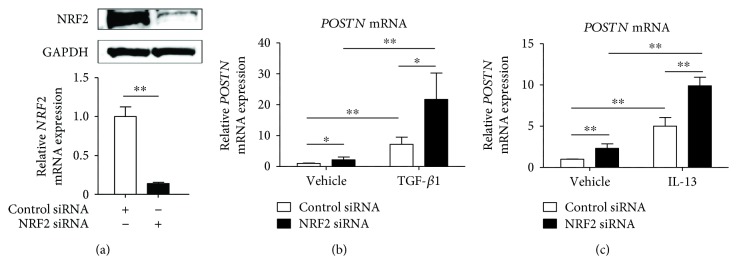
Knockdown of *NRF2* upregulates the expression of periostin in dermal fibroblasts. (a) NHDF cells were transiently transfected with control siRNA or *NRF2* siRNA for 24 h, after which whole cell lysates or total RNA were extracted. The expression levels of *NRF2* were normalized to that of *GAPDH*. NRF2 protein and mRNA expressions are depicted. (b, c) NHDF cells were transiently transfected with control siRNA or 5 nM *NRF2* siRNA for 24 h and then stimulated with 5 ng/mL TGF-*β*1 (b) or IL-13 (c). *POSTN* mRNA expression is depicted. The values were adjusted by *GAPDH* expression. The same experiments were performed three times. ^∗^*P* < 0.05, ^∗∗^*P* < 0.01.

To elucidate the role of NRF2 in baseline and cytokine-induced expression of POSTN, we next knocked down NRF2 using NRF2 siRNA. The knockdown efficiency was 86.1 ± 1.2% as assessed by qRT-PCR and was confirmed by Western blotting analysis (Figure 3(a)). Interestingly, the baseline expression of POSTN was significantly enhanced in the fibroblasts with NRF2 knockdown compared with those transfected with control siRNA (Figures 3(b) and 3(c)). In addition, both TGF-*β*1- and IL-13-mediated POSTN were further augmented in the fibroblasts with NRF2 knockdown compared with the levels in control fibroblasts (Figures 3(b) and 3(c)). Taken together, these results demonstrate that NRF2 downregulates both constitutive and inducible POSTN expression.

### 3.4. NRF2 Knockdown Abolishes CIN-Mediated Downregulation of POSTN

**Figure 4:**
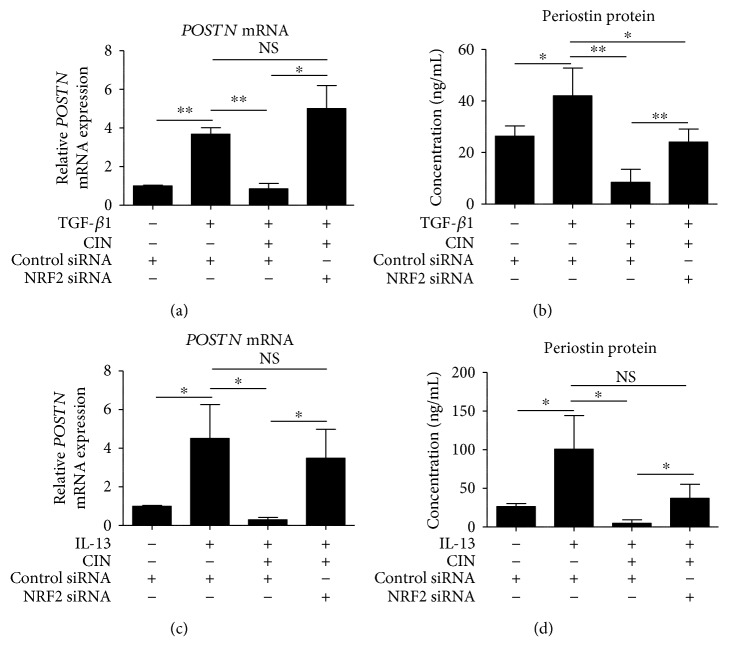
The involvement of NRF2 signaling in the downregulation of IL-13- and TGF-*β*1-induced periostin expression by cinnamaldehyde. (a, b) Knockdown of *NRF2* reverses the effects of cinnamaldehyde (CIN) on TGF-*β*1-induced expression of periostin (POSTN) mRNA and protein. (c, d) Knockdown of *NRF2* reverses the effects of CIN on IL-13-induced expression of *POSTN* mRNA and protein. NHDF cells were transiently transfected with control siRNA or *NRF2* siRNA for 24 h; next, cells were precultured in 25 *μ*M CIN for 30 min and then treated with 5 ng/mL TGF-*β*1 or 50 ng/mL IL-13 or left untreated for 48 h. Expression levels of *POSTN* mRNA and protein in the cell supernatants are depicted. The values were adjusted by *GAPDH* expression. The same experiments were performed three times. ^∗^*P* < 0.05, ^∗∗^*P* < 0.01. NS: not significant.

**Figure 5:**
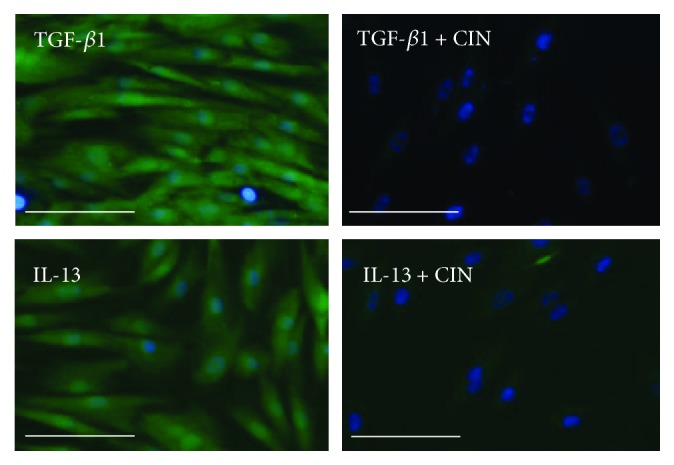
Effects of cinnamaldehyde on TGF-*β*1- and IL-13-induced ROS production in dermal fibroblasts. NHDF cells were precultured in 25 *μ*M cinnamaldehyde (CIN) for 16 h and then treated with 20 ng/mL TGF-*β*1 or 50 ng/mL IL-13 for 20 min. Cells were incubated with carboxy-H_2_DCFDA for 25 min, and ROS production was visualized by fluorescence microscopy. Scale bars, 100 *μ*m.

**Figure 6:**
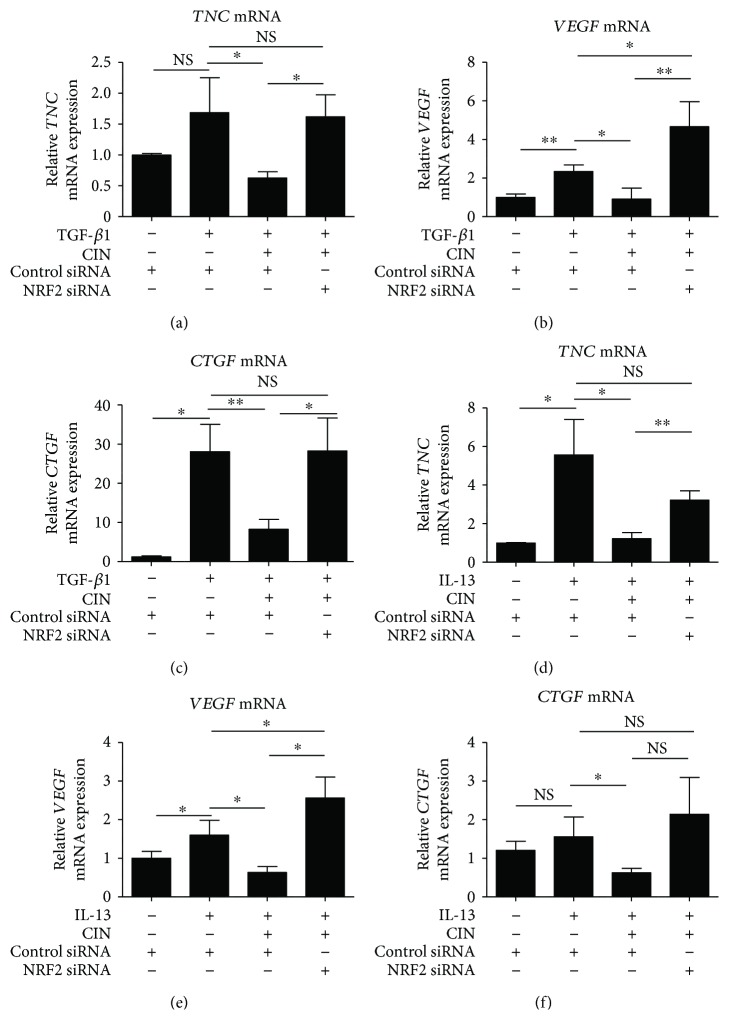
The involvement of NRF2 signaling in the downregulation of IL-13- and TGF-*β*1-induced tenascin-C, VEGF, and CTGF mRNA expression by cinnamaldehyde. (a–c) Knockdown of *NRF2* reverses the effects of cinnamaldehyde (CIN) on TGF-*β*1-induced mRNA expression of *TNC*, *VEGF*, and *CTGF*. (d–f) Knockdown of *NRF2* reverses the effects of CIN on IL-13-induced mRNA expression of *TNC*, *VEGF*, and *CTGF*. NHDF cells were transiently transfected with control siRNA or 5 nM *NRF2* siRNA for 24 h; next, cells were precultured with 25 *μ*M CIN for 30 min and then treated with 5 ng/mL TGF-*β*1 or 50 ng/mL IL-13 or left untreated for 48 h. mRNA expression levels of *TNC*, *VEGF*, and *CTGF* are depicted. The values were adjusted by *GAPDH* expression. The same experiments were performed three times. ^∗^*P* < 0.05, ^∗∗^*P* < 0.01. NS: not significant.

We next examined whether CIN-mediated downregulation of POSTN is dependent on NRF2. As shown in Figures 4(a) and 4(b), TGF-*β*1 upregulated POSTN expression at the mRNA and protein levels, which CIN in turn inhibited. The capacity of CIN to inhibit TGF-*β*1 signaling was abrogated in fibroblasts with NRF2 knockdown. Moreover, NRF2 knockdown abolished the inhibitory action of CIN on IL-13-mediated *POSTN* upregulation (Figures 4(c) and 4(d)). We next attempted to confirm that CIN actually inhibits TGF-*β*1- and IL-13-mediated oxidative stress. As expected, CIN inhibited both TGF-*β*1- and IL-13-induced production of ROS (Figure 5). In addition, we investigated the effects of CIN on TGF-*β*1- and IL-13-induced gene expression of other profibrotic mediators, namely, tenascin-C (*TNC*), vascular endothelial growth factor (*VEGF*), and connective tissue growth factor (*CTGF*). As shown in Figure 6, the expression of all *TNC*, *VEGF*, and *CTGF* was upregulated by TGF-*β*1 and IL-13, respectively, which was significantly inhibited by CIN in all cases. Notably, the CIN-mediated inhibitory capacity was again abolished in fibroblasts with NRF2 knockdown, suggesting that NRF2 signaling has multiple antifibrotic effects.

## 4. Discussion

**Figure 7:**
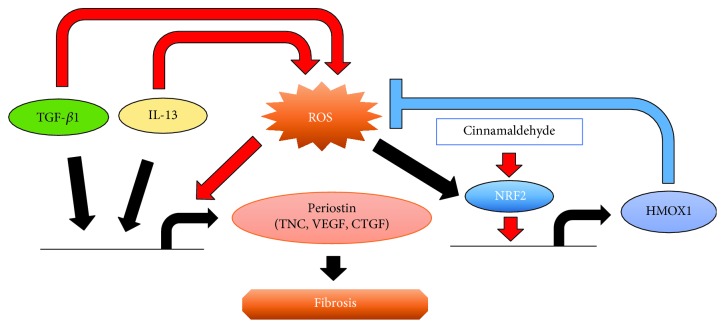
Schematic model of the molecular mechanism by which cinnamaldehyde inhibits both IL-13- and TGF-*β*1-mediated periostin expression in fibroblasts. Cinnamaldehyde induces NRF2 nuclear translocation and HMOX1 production, which downregulates IL-13- and TGF-*β*1-mediated *POSTN*, *TNC*, *VEGF*, and *CTGF* expression by reducing ROS production, followed by the attenuation of fibrosis.

Fibrosis is a complex condition mediated by profibrotic factors, an imbalance of collagen synthesis and degradation, upregulation of the extracellular matrix including POSTN and TNC, and increased oxidative stress. However, the mechanisms by which POSTN expression is regulated in fibrotic disease and their relationships with oxidative stress have remained elusive. In this study, we made the following key findings, which shed light on the mechanisms involved: (i) CIN activates NRF2 signaling and upregulates HMOX1 production in dermal fibroblasts. (ii) CIN inhibits POSTN expression induced by TGF-*β*1 and IL-13 in dermal fibroblasts. (iii) Knockdown of NRF2 upregulates mRNA expression of constitutive and TGF-*β*1- and IL-13-induced POSTN. (iv) The CIN-mediated inhibition of *POSTN* expression induced by TGF-*β*1 and IL-13 is dependent on NRF2 signaling. (v) The same kind of regulation via NRF2 signaling controls other profibrotic molecules, namely, *TNC*, *VEGF*, and *CTGF*. These findings suggest that CIN has a potential for treating fibrosis (Figure 7).

Systemic fibrosing or sclerotic disorders such as scleroderma, pulmonary fibrosis, hepatic fibrosis, renal fibrosis, and cardiac fibrosis are life-threatening, but only very limited treatment modalities are available for them [39–42]. Although new therapies such as nintedanib, pirfenidone, tocilizumab, and rapamycin are emerging [13, 18, 40, 43, 44], additional safe and tolerable medicaments are demanded.

The profibrotic process in each organ involves unopposed collagen accumulation [11, 45]. In addition to this collagen accumulation, most fibrotic lesions in systemic fibrotic diseases are associated with the upregulated expression of POSTN, which is mainly produced from activated fibroblasts [4, 6, 7, 21, 35, 46–49]. Notably, accumulated evidence indicates that stromal POSTN plays a key role in augmenting the production of collagen [48, 50] and suggests that POSTN is a potential target for the treatment of systemic fibrosis [4, 6, 7, 46].

Two different cytokines, TGF-*β*1 and IL-13, upregulate POSTN expression via distinct signaling pathways [1, 2, 9, 33, 34]. The *Kampo* herbal medicine *Keishi-bukuryo-gan* inhibits collagen production in cultured fibroblasts derived from scleroderma patients [31]. Moreover, Japanese case series with 21 patients with systemic sclerosis showed that the *Kampo* herbal medicine including Keishi-bukuryo-gan improves clinical sclerotic symptoms in 19 scleroderma patients (90.4%) [32]. Based on this background, we speculated that CIN, a major active ingredient of *Keishi-bukuryo-gan*, might downregulate POSTN expression in fibroblasts.

In this study, we indeed demonstrated that CIN inhibited POSTN expression in fibroblasts stimulated by either TGF-*β*1 or IL-13 signaling. CIN is a safe natural product derived from cinnamon, which is a commonly used spice. CIN is also known to be a potent antioxidant phytochemical that activates the antioxidant NRF2/HMOX1 and NQO1 axis [29, 51]. As TGF-*β*1 and IL-13 signaling induces oxidative stress by producing ROS [27, 52], we hypothesized that CIN/NRF2-mediated antioxidant activity contributes to the inhibitory action on both TGF-*β*1-induced and IL-13-induced *POSTN* upregulation. As expected, under our experimental conditions, CIN induced the cytoplasmic-to-nuclear translocation of NRF2 and the NRF2 activation upregulated the expression of *HMOX1* and *NQO1* at the mRNA level, but only the expression of HMOX1 at the protein level (Figure 1). Various compounds including CIN were shown to promote NRF2-mediated HMOX1 and NQO1 genes in several cells such as fibroblasts, keratinocytes, endothelial cells, epithelial colon cells, and cardiomyocytes [29, 51, 53–55]. Our present findings and previous study demonstrate that activation of cellular antioxidant response orchestrated by NRF2 has been revealed through primarily increased HMOX1 in human dermal fibroblasts [54]. The baseline as well as TGF-*β*1-induced and IL-13-induced *POSTN* upregulation was augmented in fibroblasts with NRF2 knockdown. Moreover, the CIN-induced inhibition of *POSTN* upregulation was abrogated by NRF2 knockdown upon both TGF-*β*1 and IL-13 stimulation. As a profibrotic cytokine, IL-13 participates in the fibrosis of skin and internal organs such as the liver and is considered to play an important role in inflammatory and fibrotic processes [56, 57]. Moreover, fibrosis is one of the key pathological features of airway remodeling in asthma, and the current study demonstrated that activating the NRF2 signaling pathway ameliorates ovalbumin-induced allergic airway inflammation, which is also supported by our present findings [58]. This study showed that both TGF-*β*1 and IL-13 stimulation induced the intracellular ROS production and the activated NRF2 signaling pathway suppressed both cytokine signaling in dermal fibroblasts. Although the underlying mechanisms are yet to be clarified, the ROS production by both TGF-*β*1 and IL-13 stimulation exacerbates fibrosis through upregulation of profibrotic molecules in synergism. These results suggest that the effect of TGF-*β*1 and IL-13 stimulation may be linked with the requirement of ROS production, and each signaling pathway was regulated by the NRF2/HMOX1 pathway. Furthermore, oxidative stress appeared to coordinately upregulate the expression of other profibrotic molecules such as *VEGF*, *CTGF*, and *TNC*. To the best of our knowledge, this is the first report demonstrating that the CIN/NRF2 pathway regulates POSTN expression induced by TGF-*β*1 and IL-13.

In conclusion, the results obtained here suggest that CIN is a broad inhibitor of POSTN expression covering both TGF-*β*1 and IL-13 signaling. Herbs and medicaments containing high level of CIN, such as *Keishi-bukuryo-gan*, may thus be applicable for the treatment and prevention of systemic fibrotic diseases.

## Figures and Tables

**Table 1:** Primer sequences used for real-time quantitative RT-PCR.

Target cDNA	Forward sequence	Reverse sequence
Human GAPDH	5′-TGCACCACCAACTGCTTAGC-3′	5′-GGCATGGACTGTGGTCATGAG-3′
Human POSTN	5′-CAGAGAAATCCCTCCATGAAA-3′	5′-CAGGAGCTCTTTCAAGTCTGC-3′
Human NRF2	5′-CTTGGCCTCATGATTCTGAAGTG-3′	5′-CCTGAGATGGTGACAAGGGTTGTA-3′
Human HMOX1	5′-AGTCTTCGCCCCTGTCTACT-3′	5′-GCTGGTGTGTAGGGGATGAC-3′
Human NQO1	5′-GGATTGGACCGAGCTGGAA-3′	5′-AATTGCAGTGAAGATGSSGGCAAC-3′
Human TNC	5′-GTCACCGTGTCAACCTGATG-3′	5′-GCCTGCCTTCAAGATTTCTG-3′
Human VEGFA	5′-AGGCCAGCACATAGGAGAGA-3′	5′-TTTCTTGCGCTTTCGTTTTT-3′
Human CTGF	5′-CCTGCAGGCTAGAGAAGCAG-3′	5′-TGGAGATTTTGGGAGTACGG-3′

## Data Availability

No data were used to support this study.
